# Supplementary material for: Associations Between Follicular Fluid Biomarkers and IVF/ICSI Outcomes in Normo-Ovulatory Women—A Systematic Review
Source: Biomolecules. 2025 Mar 20;15(3):443. doi: 10.3390/biom15030443 (PMC11940193; doi:10.3390/biom15030443)
Supplement: Supplementary file 1 [file biomolecules-15-00443-s001.zip › S4. NOS for cohort studies.pdf]

NEWCASTLE - OTTAWA QUALITY ASSESSMENT SCALE
COHORT STUDIES

Scores of each study (maximum score of 9):

First author, Year	Selection	Comparability	Outcome	Total score
Sun X et al., 2022	✵✵✵✵	✵✵	✵✵✵	9
Wu et al., 2012	✵✵✵✵	✵	✵✵✵	8
Habibi et al., 2022	✵✵✵✵	✵✵	✵✵✵	9
Song et al., 2019	✵✵✵✵	✵✵	✵✵✵	9
Sun Z et al., 2017	✵✵✵✵	✵✵	✵✵✵	9
Ekapatria et al., 2022	✵✵✵✵	✵	✵✵✵	8

Detailed scoring of each study can be found below.

NEWCASTLE - OTTAWA QUALITY ASSESSMENT SCALE
COHORT STUDIES

Note: A study can be awarded a maximum of one star for each numbered item within the Selection and Outcome categories. A maximum of two stars can be given for Comparability. Answers indicated by "*".

Study: Sun X et al.; Decreased histidine-rich glycoprotein and increased complement C4-B protein levels in follicular fluid predict the IVF outcomes of recurrent spontaneous abortion

Selection

1) Representativeness of the exposed cohort
   a) truly representative of the average couple with infertility in the community ❋
   *b) somewhat representative of the average couple with infertility in the community ❋
   c) selected group of users eg nurses, volunteers
   d) no description of the derivation of the cohort

2) Selection of the non exposed cohort
   *a) drawn from the same community as the exposed cohort ❋
   b) drawn from a different source
   c) no description of the derivation of the non exposed cohort

3) Ascertainment of exposure
   *a) secure record (eg surgical records) ❋
   b) structured interview ❋
   c) written self report
   d) no description

4) Demonstration that outcome of interest was not present at start of study
   *a) yes ❋
   b) no

Comparability

1) Comparability of cohorts on the basis of the design or analysis
   *a) study controls for follicular fluid levels of Histidine-rich glycoprotein (HRG) ❋
   *b) study also controls for follicular fluid levels of complement protein C4-B❋

Outcome

1) Assessment of outcome
   a) independent blind assessment ❋
   *b) record linkage ❋
   c) self report
   d) no description

2) Was follow-up long enough for outcomes to occur
   *a) yes ❋
   b) no

3) Adequacy of follow up of cohorts
   *a) complete follow up - all subjects accounted for ❋
   b) subjects lost to follow up unlikely to introduce bias - small number lost - > ____ % follow up ❋
   c) follow up rate < ____% and no description of those lost
   d) no statement

NEWCASTLE - OTTAWA QUALITY ASSESSMENT SCALE
COHORT STUDIES

Note: A study can be awarded a maximum of one star for each numbered item within the Selection and Outcome categories. A maximum of two stars can be given for Comparability. Answers indicated by "*".

Study: Wu et al.; Bone morphogenetic protein-15 in follicle fluid combined with age may differentiate between successful and unsuccessful poor ovarian responders

Selection

1) Representativeness of the exposed cohort
   a) truly representative of the average couple with infertility in the community ❋
   *b) somewhat representative of the average couple with infertility in the community ❋
   c) selected group of users eg nurses, volunteers
   d) no description of the derivation of the cohort

2) Selection of the non exposed cohort
   *a) drawn from the same community as the exposed cohort ❋
   b) drawn from a different source
   c) no description of the derivation of the non exposed cohort

3) Ascertainment of exposure
   *a) secure record (eg surgical records) ❋
   b) structured interview ❋
   c) written self report
   d) no description

4) Demonstration that outcome of interest was not present at start of study
   *a) yes ❋
   b) no

Comparability

1) Comparability of cohorts on the basis of the design or analysis
   *a) study controls for follicular fluid levels of Bone morpho-genic protein-15 (BMP-15) ❋
   b) study controls for any additional factor (None) ❋

Outcome

1) Assessment of outcome
   a) independent blind assessment ❋
   *b) record linkage ❋
   c) self report
   d) no description

2) Was follow-up long enough for outcomes to occur
   *a) yes ❋
   b) no

3) Adequacy of follow up of cohorts
   *a) complete follow up - all subjects accounted for ❋
   b) subjects lost to follow up unlikely to introduce bias - small number lost - > ____ % follow up ❋
   c) follow up rate < ____% and no description of those lost
   d) no statement

NEWCASTLE - OTTAWA QUALITY ASSESSMENT SCALE
COHORT STUDIES

Note: A study can be awarded a maximum of one star for each numbered item within the Selection and Outcome categories. A maximum of two stars can be given for Comparability. Answers indicated by "*".

Study: Habibi et al.; Expression analysis of genes and MicroRNAs involved in recurrent implantation failure: New noninvasive biomarkers of implantation

Selection

1) Representativeness of the exposed cohort
   a) truly representative of the average couple with infertility in the community ✵
   *b) somewhat representative of the average couple with infertility in the community ✵
   c) selected group of users eg nurses, volunteers
   d) no description of the derivation of the cohort

2) Selection of the non exposed cohort
   *a) drawn from the same community as the exposed cohort ✵
   b) drawn from a different source
   c) no description of the derivation of the non exposed cohort

3) Ascertainment of exposure
   *a) secure record (eg surgical records) ✵
   b) structured interview ✵
   c) written self report
   d) no description

4) Demonstration that outcome of interest was not present at start of study
   *a) yes ✵
   b) no

Comparability

1) Comparability of cohorts on the basis of the design or analysis
   *a) study controls for follicular fluid levels of Progesterone and Prostaglandin (E2) ✵
   *b) study also controls for follicular fluid levels of expression of four microRNAs ✵

Outcome

1) Assessment of outcome
   a) independent blind assessment ✵
   *b) record linkage ✵
   c) self report
   d) no description

2) Was follow-up long enough for outcomes to occur
   *a) yes ✵
   b) no

3) Adequacy of follow up of cohorts
   *a) complete follow up - all subjects accounted for ✵
   b) subjects lost to follow up unlikely to introduce bias - small number lost - > ____ % follow up ✵
   c) follow up rate < ____% and no description of those lost
   d) no statement

NEWCASTLE - OTTAWA QUALITY ASSESSMENT SCALE
COHORT STUDIES

Note: A study can be awarded a maximum of one star for each numbered item within the Selection and Outcome categories. A maximum of two stars can be given for Comparability. Answers indicated by “*”.

Study: Song et al.; Novel high-coverage targeted metabolomics method (SWATHtoMRM) for exploring follicular fluid metabolome alterations in women with recurrent spontaneous abortion undergoing in vitro fertilization

Selection

1) Representativeness of the exposed cohort
   a) truly representative of the average couple with infertility in the community ✵
   *b) somewhat representative of the average couple with infertility in the community ✵
   c) selected group of users eg nurses, volunteers
   d) no description of the derivation of the cohort

2) Selection of the non exposed cohort
   *a) drawn from the same community as the exposed cohort ✵
   b) drawn from a different source
   c) no description of the derivation of the non exposed cohort

3) Ascertainment of exposure
   *a) secure record (eg surgical records) ✵
   b) structured interview ✵
   c) written self report
   d) no description

4) Demonstration that outcome of interest was not present at start of study
   *a) yes ✵
   b) no

Comparability

1) Comparability of cohorts on the basis of the design or analysis
   *a) study controls for follicular fluid levels of 2 hormones, 3 amino acids and 2 vitamins ✵
   *b) study also controls for follicular fluid levels of Lithocholic acid and 10 lipids ✵

Outcome

1) Assessment of outcome
   a) independent blind assessment ✵
   *b) record linkage ✵
   c) self report
   d) no description

2) Was follow-up long enough for outcomes to occur
   *a) yes ✵
   b) no

3) Adequacy of follow up of cohorts
   *a) complete follow up - all subjects accounted for ✵
   b) subjects lost to follow up unlikely to introduce bias - small number lost - > ____ % follow up ✵
   c) follow up rate < ____% and no description of those lost
   d) no statement

NEWCASTLE - OTTAWA QUALITY ASSESSMENT SCALE
COHORT STUDIES

Note: A study can be awarded a maximum of one star for each numbered item within the Selection and Outcome categories. A maximum of two stars can be given for Comparability. Answers indicated by "*".

Study: Sun Z et al.; Human Follicular Fluid Metabolomics Study of Follicular Development and Oocyte Quality

Selection

1) Representativeness of the exposed cohort
   a) truly representative of the average couple with infertility in the community ✵
   *b) somewhat representative of the average couple with infertility in the community ✵
   c) selected group of users eg nurses, volunteers
   d) no description of the derivation of the cohort

2) Selection of the non exposed cohort
   *a) drawn from the same community as the exposed cohort ✵
   b) drawn from a different source
   c) no description of the derivation of the non exposed cohort

3) Ascertainment of exposure
   *a) secure record (eg surgical records) ✵
   b) structured interview ✵
   c) written self report
   d) no description

4) Demonstration that outcome of interest was not present at start of study
   *a) yes ✵
   b) no

Comparability

1) Comparability of cohorts on the basis of the design or analysis
   *a) study controls for follicular fluid levels of Deoxycorticosterone and Nicotine, 2 nucleic acid metabolites, 2 amino acid metabolites, 1 vitamin metabolite, 8 lipids ✵
   *b) study also controls for follicular fluid levels of 2 nucleic acid metabolites, 2 amino acid metabolites, 1 vitamin metabolite and 8 lipids ✵

Outcome

1) Assessment of outcome
   a) independent blind assessment ✵
   *b) record linkage ✵
   c) self report
   d) no description

2) Was follow-up long enough for outcomes to occur
   *a) yes ✵
   b) no

3) Adequacy of follow up of cohorts
   *a) complete follow up - all subjects accounted for ✵
   b) subjects lost to follow up unlikely to introduce bias - small number lost - > ____ % follow up ✵
   c) follow up rate < ____% and no description of those lost
   d) no statement

NEWCASTLE - OTTAWA QUALITY ASSESSMENT SCALE
COHORT STUDIES

Note: A study can be awarded a maximum of one star for each numbered item within the Selection and Outcome categories. A maximum of two stars can be given for Comparability. Answers indicated by “*”.

Study: Ekapatria et al.; The Effects of Follicular Fluid 25(OH)D Concentration on Intrafollicular Estradiol Level, Oocyte Quality, and Fertilization Rate in Women Who Underwent IVF Program

Selection

1) Representativeness of the exposed cohort
   a) truly representative of the average couple with infertility in the community ✸
   *b) somewhat representative of the average couple with infertility in the community ✸
   c) selected group of users eg nurses, volunteers
   d) no description of the derivation of the cohort

2) Selection of the non exposed cohort
   *a) drawn from the same community as the exposed cohort ✸
   b) drawn from a different source
   c) no description of the derivation of the non exposed cohort

3) Ascertainment of exposure
   *a) secure record (eg surgical records) ✸
   b) structured interview ✸
   c) written self report
   d) no description

4) Demonstration that outcome of interest was not present at start of study
   *a) yes ✸
   b) no

Comparability

1) Comparability of cohorts on the basis of the design or analysis
   *a) study controls for follicular fluid levels of vitamin D ✸
   b) study controls for any additional factor (None) ✸

Outcome

1) Assessment of outcome
   a) independent blind assessment ✸
   *b) record linkage ✸
   c) self report
   d) no description

2) Was follow-up long enough for outcomes to occur
   *a) yes ✸
   b) no

3) Adequacy of follow up of cohorts
   *a) complete follow up - all subjects accounted for ✸
   b) subjects lost to follow up unlikely to introduce bias - small number lost - > ____ % follow up ✸
   c) follow up rate < ____% and no description of those lost
   d) no statement
