# Supplementary material for: Associations Between Follicular Fluid Biomarkers and IVF/ICSI Outcomes in Normo-Ovulatory Women—A Systematic Review
Source: Biomolecules. 2025 Mar 20;15(3):443. doi: 10.3390/biom15030443 (PMC11940193; doi:10.3390/biom15030443)
Supplement: Supplementary file 1 [file biomolecules-15-00443-s001.zip › S5. NOS for case-control studies.pdf]

NEWCASTLE - OTTAWA QUALITY ASSESSMENT SCALE
CASE CONTROL STUDIES

Scores of each study (maximum score of 9):

First author, Year	Selection	Comparability	Outcome	Total score
Bastu et al., 2015	✸✸✸	✸✸	✸✸✸	8
Atabakhsh et al., 2018	✸✸✸	✸✸	✸✸✸	8
Bilen et al., 2014	✸✸✸	✸✸	✸✸✸	8
Mostafa et al., 2019	✸✸✸	✸✸	✸✸✸	8
Jasimkadim et al., 2020	✸✸✸	✸✸	✸✸✸	8
Mehta et al., 2013	✸✸✸	✸✸	✸✸✸	8
Celik et al., 2013	✸✸✸	✸	✸✸✸	7
Carpintero et al., 2014	✸✸✸	✸✸	✸✸✸	8
Yang et al., 2020	✸✸✸	✸✸	✸✸✸	8
Alzubaidy et al., 2021	✸✸✸	✸	✸✸✸	7
Ahmeid et al., 2017	✸✸✸	✸	✸✸✸	7
Buyuk et al., 2017	✸✸✸	✸	✸✸✸	7
Akarsu et al., 2017	✸✸✸	✸✸	✸✸✸	8
Abdul-Razzaq et al., 2020	✸✸✸	✸	✸✸✸	7
Ocal et al., 2012	✸✸✸	✸	✸✸✸	7
Gode et al., 2019	✸✸✸	✸✸	✸✸✸	8

Detailed scoring of each study can be found below.

NEWCASTLE - OTTAWA QUALITY ASSESSMENT SCALE
CASE CONTROL STUDIES

Note: A study can be awarded a maximum of one star for each numbered item within the Selection and Exposure categories. A maximum of two stars can be given for Comparability.

Study: Bastu et al.; The association between follicular fluid levels of cathepsin B, relaxin or AMH with clinical pregnancy rates in infertile patients

Selection

1) Is the case definition adequate?
   *a) yes, with independent validation ✵
   b) yes, eg record linkage or based on self reports
   c) no description

2) Representativeness of the cases
   *a) consecutive or obviously representative series of cases ✵
   b) potential for selection biases or not stated

3) Selection of Controls
   a) community controls ✵
   *b) hospital controls
   c) no description

4) Definition of Controls
   *a) no history of disease (endpoint) ✵
   b) no description of source

Comparability

1) Comparability of cases and controls on the basis of the design or analysis
   *a) study controls for fertilization rate and pregnancy rate ✵
   *b) study also controls for oocyte count and oocyte quality ✵

Exposure

1) Ascertainment of exposure
   *a) secure record (eg surgical records) ✵
   b) structured interview where blind to case/control status ✵
   c) interview not blinded to case/control status
   d) written self report or medical record only
   e) no description

2) Same method of ascertainment for cases and controls
   *a) yes ✵
   b) no

3) Non-Response rate
   *a) same rate for both groups (0%) ✵
   b) non respondents described
   c) rate different and no designation

NEWCASTLE - OTTAWA QUALITY ASSESSMENT SCALE
CASE CONTROL STUDIES

Note: A study can be awarded a maximum of one star for each numbered item within the Selection and Exposure categories. A maximum of two stars can be given for Comparability.

Study: Atabakhsh et al.; Activity of matrix metalloproteinase 2 and 9 in follicular fluid and seminal plasma and its relation to embryo quality and fertilization rate

Selection

1) Is the case definition adequate?
   *a) yes, with independent validation ✵
   b) yes, eg record linkage or based on self reports
   c) no description

2) Representativeness of the cases
   *a) consecutive or obviously representative series of cases ✵
   b) potential for selection biases or not stated

3) Selection of Controls
   a) community controls ✵
   *b) hospital controls
   c) no description

4) Definition of Controls
   *a) no history of disease (endpoint) ✵
   b) no description of source

Comparability

1) Comparability of cases and controls on the basis of the design or analysis
   *a) study controls for fertilization rate and embryo quality ✵
   *b) study also controls for oocyte count and oocyte quality ✵

Exposure

1) Ascertainment of exposure
   *a) secure record (eg surgical records) ✵
   b) structured interview where blind to case/control status ✵
   c) interview not blinded to case/control status
   d) written self report or medical record only
   e) no description

2) Same method of ascertainment for cases and controls
   *a) yes ✵
   b) no

3) Non-Response rate
   *a) same rate for both groups (0%) ✵
   b) non respondents described
   c) rate different and no designation

NEWCASTLE - OTTAWA QUALITY ASSESSMENT SCALE
CASE CONTROL STUDIES

Note: A study can be awarded a maximum of one star for each numbered item within the Selection and Exposure categories. A maximum of two stars can be given for Comparability.

Study: Bilen et al.; Do follicular fluid gelatinase levels affect fertilization rates and oocyte quality?

Selection

1) Is the case definition adequate?
   *a) yes, with independent validation ✵
   b) yes, eg record linkage or based on self reports
   c) no description

2) Representativeness of the cases
   *a) consecutive or obviously representative series of cases ✵
   b) potential for selection biases or not stated

3) Selection of Controls
   a) community controls ✵
   *b) hospital controls
   c) no description

4) Definition of Controls
   *a) no history of disease (endpoint) ✵
   b) no description of source

Comparability

1) Comparability of cases and controls on the basis of the design or analysis
   *a) study controls for fertilization rate ✵
   *b) study also controls for oocyte count and oocyte quality ✵

Exposure

1) Ascertainment of exposure
   *a) secure record (eg surgical records) ✵
   b) structured interview where blind to case/control status ✵
   c) interview not blinded to case/control status
   d) written self report or medical record only
   e) no description

2) Same method of ascertainment for cases and controls
   *a) yes ✵
   b) no

3) Non-Response rate
   *a) same rate for both groups (0%) ✵
   b) non respondents described
   c) rate different and no designation

NEWCASTLE - OTTAWA QUALITY ASSESSMENT SCALE
CASE CONTROL STUDIES

Note: A study can be awarded a maximum of one star for each numbered item within the Selection and Exposure categories. A maximum of two stars can be given for Comparability.

Study: Mostafa et al.; Effect of Follicular Fluid Lactoferrin Level on Oocytes Quality and Pregnancy Rate in Intracytoplasmic Sperm Injection Cycles

Selection

1) Is the case definition adequate?
   *a) yes, with independent validation ✸
   b) yes, eg record linkage or based on self reports
   c) no description

2) Representativeness of the cases
   *a) consecutive or obviously representative series of cases ✸
   b) potential for selection biases or not stated

3) Selection of Controls
   a) community controls ✸
   *b) hospital controls
   c) no description

4) Definition of Controls
   *a) no history of disease (endpoint) ✸
   b) no description of source

Comparability

1) Comparability of cases and controls on the basis of the design or analysis
   *a) study controls for fertilization rate, embryo quality and pregnancy rate ✸
   *b) study also controls for oocyte count and oocyte quality ✸

Exposure

1) Ascertainment of exposure
   *a) secure record (eg surgical records) ✸
   b) structured interview where blind to case/control status ✸
   c) interview not blinded to case/control status
   d) written self report or medical record only
   e) no description

2) Same method of ascertainment for cases and controls
   *a) yes ✸
   b) no

3) Non-Response rate
   *a) same rate for both groups (0%) ✸
   b) non respondents described
   c) rate different and no designation

NEWCASTLE - OTTAWA QUALITY ASSESSMENT SCALE
CASE CONTROL STUDIES

Note: A study can be awarded a maximum of one star for each numbered item within the Selection and Exposure categories. A maximum of two stars can be given for Comparability.

Study: Jasimkadim et al.; Concentration of R-spondin 2 in the Follicular Fluid is Correlated with Implantation Rate, Estrogen and Amphiregulin, in Iraqi Women Undergo ICSI

Selection

1) Is the case definition adequate?
   *a) yes, with independent validation ✵
   b) yes, eg record linkage or based on self reports
   c) no description

2) Representativeness of the cases
   *a) consecutive or obviously representative series of cases ✵
   b) potential for selection biases or not stated

3) Selection of Controls
   a) community controls ✵
   *b) hospital controls
   c) no description

4) Definition of Controls
   *a) no history of disease (endpoint) ✵
   b) no description of source

Comparability

1) Comparability of cases and controls on the basis of the design or analysis
   *a) study controls for pregnancy rate ✵
   *b) study also controls for implantation rate ✵

Exposure

1) Ascertainment of exposure
   *a) secure record (eg surgical records) ✵
   b) structured interview where blind to case/control status ✵
   c) interview not blinded to case/control status
   d) written self report or medical record only
   e) no description

2) Same method of ascertainment for cases and controls
   *a) yes ✵
   b) no

3) Non-Response rate
   *a) same rate for both groups (0%) ✵
   b) non respondents described
   c) rate different and no designation

NEWCASTLE - OTTAWA QUALITY ASSESSMENT SCALE
CASE CONTROL STUDIES

Note: A study can be awarded a maximum of one star for each numbered item within the Selection and Exposure categories. A maximum of two stars can be given for Comparability.

Study: Mehta et al.; Follicular fluid insulin like growth factor-1 (FF IGF-1) is a biochemical marker of embryo quality and implantation rates in in vitro fertilization cycles.

Selection

1) Is the case definition adequate?
   *a) yes, with independent validation ✵
   b) yes, eg record linkage or based on self reports
   c) no description

2) Representativeness of the cases
   *a) consecutive or obviously representative series of cases ✵
   b) potential for selection biases or not stated

3) Selection of Controls
   a) community controls ✵
   *b) hospital controls
   c) no description

4) Definition of Controls
   *a) no history of disease (endpoint) ✵
   b) no description of source

Comparability

1) Comparability of cases and controls on the basis of the design or analysis
   *a) study controls for pregnancy rate ✵
   *b) study also controls for embryo quality and implantation rate ✵

Exposure

1) Ascertainment of exposure
   *a) secure record (eg surgical records) ✵
   b) structured interview where blind to case/control status ✵
   c) interview not blinded to case/control status
   d) written self report or medical record only
   e) no description

2) Same method of ascertainment for cases and controls
   *a) yes ✵
   b) no

3) Non-Response rate
   *a) same rate for both groups (0%) ✵
   b) non respondents described
   c) rate different and no designation

NEWCASTLE - OTTAWA QUALITY ASSESSMENT SCALE
CASE CONTROL STUDIES

Note: A study can be awarded a maximum of one star for each numbered item within the Selection and Exposure categories. A maximum of two stars can be given for Comparability.

Study: Celik et al.; Effect of ovarian stimulation with recombinant follicle-stimulating hormone, gonadotropin-releasing hormone agonist and antagonists, on follicular fluid stem cell factor and serum urocortin 1 levels on the day of oocyte retrieval

Selection

1) Is the case definition adequate?
   *a) yes, with independent validation ✵
   b) yes, eg record linkage or based on self reports
   c) no description

2) Representativeness of the cases
   *a) consecutive or obviously representative series of cases ✵
   b) potential for selection biases or not stated

3) Selection of Controls
   a) community controls ✵
   *b) hospital controls
   c) no description

4) Definition of Controls
   *a) no history of disease (endpoint) ✵
   b) no description of source

Comparability

1) Comparability of cases and controls on the basis of the design or analysis
   *a) study controls for pregnancy rate ✵
   b) study controls for any additional factor (None) ✵

Exposure

1) Ascertainment of exposure
   *a) secure record (eg surgical records) ✵
   b) structured interview where blind to case/control status ✵
   c) interview not blinded to case/control status
   d) written self report or medical record only
   e) no description

2) Same method of ascertainment for cases and controls
   *a) yes ✵
   b) no

3) Non-Response rate
   *a) same rate for both groups (0%) ✵
   b) non respondents described
   c) rate different and no designation

NEWCASTLE - OTTAWA QUALITY ASSESSMENT SCALE
CASE CONTROL STUDIES

Note: A study can be awarded a maximum of one star for each numbered item within the Selection and Exposure categories. A maximum of two stars can be given for Comparability.

Study: Carpintero et al.; Follicular steroid hormones as markers of oocyte quality and oocyte development potential

Selection

1) Is the case definition adequate?
   *a) yes, with independent validation ✸
   b) yes, eg record linkage or based on self reports
   c) no description

2) Representativeness of the cases
   *a) consecutive or obviously representative series of cases ✸
   b) potential for selection biases or not stated

3) Selection of Controls
   a) community controls ✸
   *b) hospital controls
   c) no description

4) Definition of Controls
   *a) no history of disease (endpoint) ✸
   b) no description of source

Comparability

1) Comparability of cases and controls on the basis of the design or analysis
   *a) study controls for pregnancy rate ✸
   *b) study also controls for oocyte quality, fertilization rate and embryo quality ✸

Exposure

1) Ascertainment of exposure
   *a) secure record (eg surgical records) ✸
   b) structured interview where blind to case/control status ✸
   c) interview not blinded to case/control status
   d) written self report or medical record only
   e) no description

2) Same method of ascertainment for cases and controls
   *a) yes ✸
   b) no

3) Non-Response rate
   *a) same rate for both groups (0%) ✸
   b) non respondents described
   c) rate different and no designation

NEWCASTLE - OTTAWA QUALITY ASSESSMENT SCALE
CASE CONTROL STUDIES

Note: A study can be awarded a maximum of one star for each numbered item within the Selection and Exposure categories. A maximum of two stars can be given for Comparability.

Study: Yang et al.; Interleukin 6 in follicular fluid reduces embryo fragmentation and improves the clinical pregnancy rate

Selection

1) Is the case definition adequate?
   *a) yes, with independent validation ✵
   b) yes, eg record linkage or based on self reports
   c) no description

2) Representativeness of the cases
   *a) consecutive or obviously representative series of cases ✵
   b) potential for selection biases or not stated

3) Selection of Controls
   a) community controls ✵
   *b) hospital controls
   c) no description

4) Definition of Controls
   *a) no history of disease (endpoint) ✵
   b) no description of source

Comparability

1) Comparability of cases and controls on the basis of the design or analysis
   *a) study controls for pregnancy rate ✵
   *b) study also controls for oocyte count, oocyte quality and embryo quality ✵

Exposure

1) Ascertainment of exposure
   *a) secure record (eg surgical records) ✵
   b) structured interview where blind to case/control status ✵
   c) interview not blinded to case/control status
   d) written self report or medical record only
   e) no description

2) Same method of ascertainment for cases and controls
   *a) yes ✵
   b) no

3) Non-Response rate
   *a) same rate for both groups (0%) ✵
   b) non respondents described
   c) rate different and no designation

NEWCASTLE - OTTAWA QUALITY ASSESSMENT SCALE
CASE CONTROL STUDIES

Note: A study can be awarded a maximum of one star for each numbered item within the Selection and Exposure categories. A maximum of two stars can be given for Comparability.

Study: Alzubaidy et al.; The Correlation between Follicular Fluid Levels of Progesterone and Anti-Müllerian Hormone and Pregnancy Rate in ICSI-Cycle

Selection

1) Is the case definition adequate?
   *a) yes, with independent validation ✵
   b) yes, eg record linkage or based on self reports
   c) no description

2) Representativeness of the cases
   *a) consecutive or obviously representative series of cases ✵
   b) potential for selection biases or not stated

3) Selection of Controls
   a) community controls ✵
   *b) hospital controls
   c) no description

4) Definition of Controls
   *a) no history of disease (endpoint) ✵
   b) no description of source

Comparability

1) Comparability of cases and controls on the basis of the design or analysis
   *a) study controls for pregnancy rate ✵
   b) study controls for any additional factor (None) ✵

Exposure

1) Ascertainment of exposure
   *a) secure record (eg surgical records) ✵
   b) structured interview where blind to case/control status ✵
   c) interview not blinded to case/control status
   d) written self report or medical record only
   e) no description

2) Same method of ascertainment for cases and controls
   *a) yes ✵
   b) no

3) Non-Response rate
   *a) same rate for both groups (0%) ✵
   b) non respondents described
   c) rate different and no designation

NEWCASTLE - OTTAWA QUALITY ASSESSMENT SCALE
CASE CONTROL STUDIES

Note: A study can be awarded a maximum of one star for each numbered item within the Selection and Exposure categories. A maximum of two stars can be given for Comparability.

Study: Ahmeid et al.; Correlation between follicular fluid leptin and the pregnancy rate in women who underwent ICSI

Selection

1) Is the case definition adequate?
   *a) yes, with independent validation ✵
   b) yes, eg record linkage or based on self reports
   c) no description

2) Representativeness of the cases
   *a) consecutive or obviously representative series of cases ✵
   b) potential for selection biases or not stated

3) Selection of Controls
   a) community controls ✵
   *b) hospital controls
   c) no description

4) Definition of Controls
   *a) no history of disease (endpoint) ✵
   b) no description of source

Comparability

1) Comparability of cases and controls on the basis of the design or analysis
   *a) study controls for pregnancy rate ✵
   b) study controls for any additional factor (None) ✵

Exposure

1) Ascertainment of exposure
   *a) secure record (eg surgical records) ✵
   b) structured interview where blind to case/control status ✵
   c) interview not blinded to case/control status
   d) written self report or medical record only
   e) no description

2) Same method of ascertainment for cases and controls
   *a) yes ✵
   b) no

3) Non-Response rate
   *a) same rate for both groups (0%) ✵
   b) non respondents described
   c) rate different and no designation

NEWCASTLE - OTTAWA QUALITY ASSESSMENT SCALE
CASE CONTROL STUDIES

Note: A study can be awarded a maximum of one star for each numbered item within the Selection and Exposure categories. A maximum of two stars can be given for Comparability.

Study: Buyuk et al.; Serum and follicular fluid monocyte chemotactic protein-1 levels are elevated in obese women and are associated with poorer clinical pregnancy rate after in vitro fertilization: a pilot study

Selection

1) Is the case definition adequate?
   *a) yes, with independent validation ✵
   b) yes, eg record linkage or based on self reports
   c) no description

2) Representativeness of the cases
   *a) consecutive or obviously representative series of cases ✵
   b) potential for selection biases or not stated

3) Selection of Controls
   a) community controls ✵
   *b) hospital controls
   c) no description

4) Definition of Controls
   *a) no history of disease (endpoint) ✵
   b) no description of source

Comparability

1) Comparability of cases and controls on the basis of the design or analysis
   *a) study controls for pregnancy rate ✵
   b) study controls for any additional factor (None) ✵

Exposure

1) Ascertainment of exposure
   *a) secure record (eg surgical records) ✵
   b) structured interview where blind to case/control status ✵
   c) interview not blinded to case/control status
   d) written self report or medical record only
   e) no description

2) Same method of ascertainment for cases and controls
   *a) yes ✵
   b) no

3) Non-Response rate
   *a) same rate for both groups (0%) ✵
   b) non respondents described
   c) rate different and no designation

NEWCASTLE - OTTAWA QUALITY ASSESSMENT SCALE
CASE CONTROL STUDIES

Note: A study can be awarded a maximum of one star for each numbered item within the Selection and Exposure categories. A maximum of two stars can be given for Comparability.

Study: Akarsu et al.; The association between coenzyme Q10 concentrations in follicular fluid with embryo morphokinetics and pregnancy rate in assisted reproductive techniques

Selection

1) Is the case definition adequate?
   *a) yes, with independent validation ✸
   b) yes, eg record linkage or based on self reports
   c) no description

2) Representativeness of the cases
   *a) consecutive or obviously representative series of cases ✸
   b) potential for selection biases or not stated

3) Selection of Controls
   a) community controls ✸
   *b) hospital controls
   c) no description

4) Definition of Controls
   *a) no history of disease (endpoint) ✸
   b) no description of source

Comparability

1) Comparability of cases and controls on the basis of the design or analysis
   *a) study controls for pregnancy rate ✸
   *b) study also controls for embryo quality ✸

Exposure

1) Ascertainment of exposure
   *a) secure record (eg surgical records) ✸
   b) structured interview where blind to case/control status ✸
   c) interview not blinded to case/control status
   d) written self report or medical record only
   e) no description

2) Same method of ascertainment for cases and controls
   *a) yes ✸
   b) no

3) Non-Response rate
   *a) same rate for both groups (0%) ✸
   b) non respondents described
   c) rate different and no designation

NEWCASTLE - OTTAWA QUALITY ASSESSMENT SCALE
CASE CONTROL STUDIES

Note: A study can be awarded a maximum of one star for each numbered item within the Selection and Exposure categories. A maximum of two stars can be given for Comparability.

Study: Abdul-Razzaq et al.; Evaluation of Total Antioxidant Capacity in Serum and Follicular Fluid of Women Undergoing ICSI and its Association with Implantation Failure

Selection

1) Is the case definition adequate?
   *a) yes, with independent validation ✵
   b) yes, eg record linkage or based on self reports
   c) no description

2) Representativeness of the cases
   *a) consecutive or obviously representative series of cases ✵
   b) potential for selection biases or not stated

3) Selection of Controls
   a) community controls ✵
   *b) hospital controls
   c) no description

4) Definition of Controls
   *a) no history of disease (endpoint) ✵
   b) no description of source

Comparability

1) Comparability of cases and controls on the basis of the design or analysis
   *a) study controls for pregnancy rate ✵
   b) study controls for any additional factor (None) ✵

Exposure

1) Ascertainment of exposure
   *a) secure record (eg surgical records) ✵
   b) structured interview where blind to case/control status ✵
   c) interview not blinded to case/control status
   d) written self report or medical record only
   e) no description

2) Same method of ascertainment for cases and controls
   *a) yes ✵
   b) no

3) Non-Response rate
   *a) same rate for both groups (0%) ✵
   b) non respondents described
   c) rate different and no designation

NEWCASTLE - OTTAWA QUALITY ASSESSMENT SCALE
CASE CONTROL STUDIES

Note: A study can be awarded a maximum of one star for each numbered item within the Selection and Exposure categories. A maximum of two stars can be given for Comparability.

Study: Ocal et al.; The association between homocysteine in the follicular fluid with embryo quality and pregnancy rate in assisted reproductive techniques

Selection

1) Is the case definition adequate?
   *a) yes, with independent validation ✵
   b) yes, eg record linkage or based on self reports
   c) no description

2) Representativeness of the cases
   *a) consecutive or obviously representative series of cases ✵
   b) potential for selection biases or not stated

3) Selection of Controls
   a) community controls ✵
   *b) hospital controls
   c) no description

4) Definition of Controls
   *a) no history of disease (endpoint) ✵
   b) no description of source

Comparability

1) Comparability of cases and controls on the basis of the design or analysis
   *a) study controls for pregnancy rate ✵
   b) study controls for any additional factor (None) ✵

Exposure

1) Ascertainment of exposure
   *a) secure record (eg surgical records) ✵
   b) structured interview where blind to case/control status ✵
   c) interview not blinded to case/control status
   d) written self report or medical record only
   e) no description

2) Same method of ascertainment for cases and controls
   *a) yes ✵
   b) no

3) Non-Response rate
   *a) same rate for both groups (0%) ✵
   b) non respondents described
   c) rate different and no designation

NEWCASTLE - OTTAWA QUALITY ASSESSMENT SCALE
CASE CONTROL STUDIES

Note: A study can be awarded a maximum of one star for each numbered item within the Selection and Exposure categories. A maximum of two stars can be given for Comparability.

Study: Gode et al.; The effect follicular fluid vitamin A, E, D and B6 on embryo morphokinetics and pregnancy rates in patients receiving assisted reproduction

Selection

1) Is the case definition adequate?
   *a) yes, with independent validation ✵
   b) yes, eg record linkage or based on self reports
   c) no description

2) Representativeness of the cases
   *a) consecutive or obviously representative series of cases ✵
   b) potential for selection biases or not stated

3) Selection of Controls
   a) community controls ✵
   *b) hospital controls
   c) no description

4) Definition of Controls
   *a) no history of disease (endpoint) ✵
   b) no description of source

Comparability

1) Comparability of cases and controls on the basis of the design or analysis
   *a) study controls for pregnancy rate ✵
   *b) study also controls for embryo quality ✵

Exposure

1) Ascertainment of exposure
   *a) secure record (eg surgical records) ✵
   b) structured interview where blind to case/control status ✵
   c) interview not blinded to case/control status
   d) written self report or medical record only
   e) no description

2) Same method of ascertainment for cases and controls
   *a) yes ✵
   b) no

3) Non-Response rate
   *a) same rate for both groups (0%) ✵
   b) non respondents described
   c) rate different and no designation
