# Supplementary material for: Associations Between Follicular Fluid Biomarkers and IVF/ICSI Outcomes in Normo-Ovulatory Women—A Systematic Review
Source: Biomolecules. 2025 Mar 20;15(3):443. doi: 10.3390/biom15030443 (PMC11940193; doi:10.3390/biom15030443)
Supplement: Supplementary file 1 [file biomolecules-15-00443-s001.zip › S6. Supplementary Tables.pdf]

Supplementary Tables

Supplementary Table S1. Studies on the effects of proteins on IVF/ICSI outcomes in normo-ovula-tory women.

Authors, year, study design	Participants	Age (years)	FF biomarker(s)	IVF/ICSI outcome(s)	Results
Xiaohe Sun et al., 2022, Cohort [1]	87 women, 43 with a history of recurrent pregnancy loss and 44 control group. ELISA analysis done on all women, TMT (tandem mass tag) analysis done only on 6 (3 pregnancy loss and 3 control)	ELISA analysis:<br>- Control group: 33 ± 0.65<br>- pregnancy loss group: 35 ± 0.8<br><br>TMT analysis:<br>- Control group: 38 ± 1 years<br>- pregnancy loss group: 37 ± 0.7	Complement protein C4B, Histidine-rich glycoproteins (HRGs)	Implantation rate	- Lower FF C4B levels and higher FF HRG levels correlate with higher implantation rates (p = 0.011, < 0.001)<br>- Higher HRG levels (56 vs 39 mg/dl) and lower C4B levels (2235 vs 2948 ng/ml) in women with successful implantation<br>- Threshold levels for implantation success of > 35.8 mg/dl for HRG and < 2739.7 ng/ml for C4B
Ercan Bastu et al., 2015, Case control [2]	79 women, 24 achieved pregnancy and 56 didn't	- Pregnant: 31.55 ± 4.76<br>- Non-pregnant: 33.12 ± 4.67	Cathepsin B	Oocyte count, oocyte quality, fertilization rate, pregnancy rate	- Positive correlation between FF cathepsin B levels and the number of retrieved oocytes, the number of MII oocytes, MII oocyte rate, fertilization rate and pregnancy rate (p = 0.033, 0.037, 0.032, 0.042, < 0.001)<br>- Higher FF Cathepsin B levels in women who achieved pregnancy (0.20 vs 0.12 μg/g)<br>- Pregnancy threshold level of > 0.12 μg/g
Mojgan Atabakhsh et al., 2018, Case control [3]	74 women	23 - 35 years	Matrix metalloproteinase 2 (MMP-2), Matrix metalloproteinase 9 (MMP-9)	Oocyte count, oocyte quality, embryo quality, fertilization rate	- Positive correlation between MMP-2 activity with oocyte quality and embryo quality (p = 0.021, 0.014)<br>- No correlation between MMP-2 activity and oocyte count or fertilization rate<br>- Positive correlation between MMP-9 activity and oocyte quality (p = 0.014)<br>- No correlation between MMP-9 activity and oocyte count, embryo quality or fertilization rate

Erdal Bilen et al., 2014, Case control [4]	60 women with unexplained infertility, 30 were given GnRH agonist and 30 were given GnRH antagonist	- given GnRH agonist: 32.8 ± 4.8<br>- given GnRH antagonist: 29.7 ± 4	Gelatinase metalloproteinase 2 (MMP-2), Gelatinase metalloproteinase 9 (MMP-9)	Oocyte count, oocyte quality, fertilization rate	- No correlation between MMP-2 and oocyte quality, fertilization rate or number of retrieved oocytes (p = 0.9)<br>- Positive correlation between MMP-9 with oocyte quality and fertilization rate (p = 0.01, 0.02)<br>- No correlation between MMP-9 and oocyte count (p = 0.5)
Mohamed Hussein Mostafa et al., 2019, Case control [5]	64 women, 44 achieved pregnancy and 20 didn't	- Pregnant: 30.7 ± 4.0<br>- Non-pregnant: 30.8 ± 4.6	Lactoferrin	Oocyte count, oocyte quality, fertilization rate, embryo quality, chemical pregnancy rate	- No significant difference in FF lactoferrin levels between women with positive chemical pregnancy and negative chemical pregnancy (0.6 vs 1.5 ng/ml, p = 0.636)<br>- No correlation between FF lactoferrin levels with the number of retrieved, mature or fertilized oocytes (p = 0.690, 0.431, 0.581)<br>- No correlation between FF lactoferrin levels with embryo quality

Supplementary Table S2. Studies on the effects of growth factors on IVF/ICSI outcomes in normo-ovulatory women.

Authors, year, study design	Participants	Age (years)	FF biomarker(s)	IVF/ICSI outcome(s)	Results
Enas Jasimkadim et al., 2020, Case control [6]	45 women, 12 achieved pregnancy and 33 didn't	18 - 42	R-spondin2, Amphiregulin (AREG), Bone morpho-genic protein-15 (BMP-15)	Implantation rate, pregnancy rate	- Significant correlation between FF R-spondin2 and FF Amphiregulin levels with implantation rates (p = 0.015, 0.008)<br>- No correlation between FF BMP-15 levels and implantation rates (p = 0.945)<br>- No significant difference in FF R-spondin2, FF Amphiregulin or FF BMP-15 levels according to pregnancy rate (p = 0.283, 0.308, 0.586)

Yan-Ting Wu et al., 2012, Cohort [7]	414 women, 207 poor COH responders and 207 normal responders split into groups according to BMP-15 level and age	- All women: < 40 years<br>- Young age group: ≤ 35<br>- Old age group: > 35	Bone morphogenic protein-15 (BMP-15)	Implantation rate, clinical pregnancy rate, chemical pregnancy rate, live birth rate	Higher BMP-15 levels (> 1.17 ng/ml) correlated with implantation rate, clinical pregnancy rate, chemical pregnancy rate and live birth rate (p = 0.023, 0.016, 0.011, 0.019)
Bindu Mehta et al., 2013, Case control [8]	120 normo-ovulatory women	- Pregnant: 31.92 ± 3.83<br>- Non-pregnant: 31.86 ± 4.35	Insulin-like growth factor-1 (IGF-1)	Embryo quality, implantation rate and clinical pregnancy rate	- Higher IGF-1 levels (> 59.25 ng/mg) in the pregnant group (130.5 vs 62.64 ng/mg, p = 0.0002)<br>- Threshold for pregnancy > 58.50 ng/mg<br>- Higher embryo quality, implantation rates and clinical pregnancy rates with higher IGF-1 levels (p < 0.0001, = 0.0152, 0.0272)
Onder Celik et al., 2013, Case control [9]	42 women on either GnRH agonist (n = 22) or GnRH antagonist (n = 20). 14 achieved pregnancy (7 in each group) and 28 didn't (15 in agonist group, 13 in antagonist group)	- given GnRH agonist: 30.2 ± 4.8<br>- given GnRH antagonist: 33.8 ± 4.3	Stem cell factor (SCF)	Pregnancy rate	FF SCF levels were similar in women who became pregnant compared to those who didn't (p = 0.71)

Supplementary Table S3. Studies on the effects of steroid hormones on IVF/ICSI outcomes in normo-ovulatory women.

**Authors, year, study design**	**Participants**	**Age (years)**	**FF biomarker(s)**	**IVF/ICSI outcome(s)**	**Results**
Nayara López Carpintero et al., 2014, Case control [10]	31 women, 11 achieved pregnancy, 4 had an abortion and 22 didn't achieve pregnancy.	< 38	Progesterone, Testosterone, Estradiol (E2), Dehydroepiandrosterone sulfate (DHEAS)	Oocyte maturation, fertilization results, embryo quality, pregnancy rates	- Levels of progesterone, testosterone, E2 and DHEAS were higher in normal fertilization cases than cases of failed fertilization, but only progesterone was significant (p = 0.003, 0.07, 0.15, 0.1)

					- Higher FF Progesterone levels in successful fertilization cases (22.5 vs 17.4 µg/ml)<br>- Higher E2 levels, E2/progesterone ratio and E2/testosterone ratio in high-quality embryos compared to low-quality embryos ($p = 0.01$, < 0.005, $= 0.001$)<br>- Higher E2 levels in women who achieved pregnancy compared to women who didn't ($p = 0.02$)
Jie Yang et al., 2020, Case control [11]	103 women suffering from tubal infertility. 72 received an embryo transfer, 48 achieved clinical pregnancy and 24 didn't.	- All women: 31.47 ± 4.07<br>- Pregnant: 31.08 ± 4.22<br>- Non-pregnant: 32.75 ± 3.73	Estradiol (E2)	Clinical pregnancy rate	No significant difference in E2 levels between pregnant and non-pregnant women (3925.25 vs 3754.76 pg/ml, $p = 0.99$)
Bahareh Habibi et al., 2022, Cohort [12]	70 women, 51 with history of RIF and 19 controls. 19 of RIF group achieved pregnancy and 32 didn't.	< 38	Progesterone	Clinical pregnancy rate	Higher progesterone levels in women who achieved pregnancy compared to women who didn't (42.48 vs 30.83 pg/ml, $p < 0.0001$), compared to control group (36.6 pg/ml)
Huda Alzubaidy et al., 2021, Case control [13]	50 women, 33 achieved pregnancy and 13 didn't	20 - 40	Progesterone	Chemical pregnancy rate	Higher progesterone levels in women who achieved pregnancy compared to women who didn't (6.81 Vs 3.38 pg/ml, $p < 0.0001$)
Jingyan Song et al., 2019, Cohort [14]	44 women were divided into pregnancy loss group (n = 22) and the control group (n = 22)	- pregnancy loss group: 29.7 ± 3.8,<br>- Control group: 30.3 ± 3.5	Dehydroepiandrosterone sulfate (DHEAS), 25-hydroxycholesterol (25-HC)	Recurrent pregnancy loss rate	Higher DHEAS and lower 25-HC levels in pregnancy loss group compared to control ($p = 0.0019$, 0.0069)
Zhengao Sun et al., 2017, Cohort [15]	48 women, 24 aged 23 - 28 years and 24 aged 35 - 46	- Younger age group: 23 - 28<br>- Older age group: 35 - 46	Deoxycorticosterone (DOC)	Oocyte quality	FF DOC was negatively correlated with oocyte quality ($p = 0.04$)

Supplementary Table S4. Studies on the effects of polypeptide hormones on IVF/ICSI outcomes in normo-ovulatory women.

Authors, year, study design	Participants	Age (years)	FF biomarker(s)	IVF/ICSI outcome(s)	Results
Huda Alzubaidy et al., 2021, Case control [13]	50 women, 33 achieved pregnancy and 13 didn't	20 - 40	Anti-mullerian hormone (AMH)	Chemical pregnancy rate	Higher FF AMH levels in women who achieved pregnancy compared to women who didn't (3.24 vs 1.16 pg/ml, $p < 0.0001$)
Ercan Bastu et al., 2015, Case control [2]	79 women, 24 achieved pregnancy and 56 didn't	- Pregnant: 31.55 ± 4.76<br>- Non-pregnant: 33.12 ± 4.67	Anti-mullerian hormone (AMH), Relaxin	Oocyte count, oocyte quality, pregnancy rate, fertilization rate	- No correlation between FF AMH levels with pregnancy rate, the number of retrieved oocytes, the number of MII oocytes, MII oocyte rate or fertilization rate (p = 0.331, 0.304, 0.306, 0.294, 0.488)<br>- No correlation between FF relaxin levels and pregnancy rate, the number of retrieved oocytes, the number of MII oocytes, MII oocyte rate or fertilization rate (p = 0.562, 0.519, 0.361, 0.504, 0.258)
Mutaz Sabah Ahmeid et al., 2017, Case control [16]	54 women, 11 achieved pregnancy and 43 didn't	16 - 44	Leptin	Pregnancy rate	FF leptin levels weren't different between women who achieved pregnancy and women who didn't (65.12 vs 50.44 ng/dl, $p < 0.05$)

Supplementary Table S5. Studies on the effects of inflammation and oxidative stress markers on IVF/ICSI outcomes in normo-ovulatory women.

Authors, year, study design	Participants	Age (years)	FF biomarker(s)	IVF/ICSI outcome(s)	Results
Jie Yang et al., 2020, Case control [11]	103 women suffering from tubal infertility. 72 received an embryo transfer, 48 achieved clinical pregnancy and 24 didn't	- All women: 31.47 ± 4.07<br>- Pregnant: 31.08 ± 4.22<br>- Non-pregnant: 32.75 ± 3.73	Interleukin 6 (IL-6)	Oocyte count, oocyte quality, embryo quality, clinical pregnancy rate	- FF IL-6 levels weren't correlated with number of retrieved oocytes or mature oocytes (p = 0.1815 and = 0.9504)<br>- Higher FF IL-6 levels with lower embryo fragmentation rates ($p < 0.0001$)<br>- No correlation between FF IL-6 and blastomere cell count or symmetry (p = 0.314 and = 0.143)<br>- Higher FF IL-6 levels in women who achieved pregnancy

					compared to women who didn't (8.39 vs 4.64 pg/ml, p = 0.016)
Erkan Buyuk, M.D. et al., 2017, Case control [17]	38 women, 11 achieved clinical pregnancy and 27 didn't. (mean BMI over 26.1)	- Pregnant: 34.7 ± 1.0<br>- Non-pregnant: 38.4 ± 0.8	Monocyte chemotactic protein-1 (MCP-1)	Clinical pregnancy rate	No correlation between FF MCP-1 levels and clinical pregnancy rates ($p < 0.05$)
Suleyman Akarsu et al., 2017, Case control [18]	60 women with unexplained infertility	- Pregnant: 31.82 ± 4.35<br>- Non-pregnant: 32.56 ± 4.92	Coenzyme Q10 (CoQ10)	Embryo quality, embryo morphokinetics, pregnancy rate	- FF CoQ10 levels were higher in high-quality embryos (0.526 vs 0.390 μg/ml, p = 0.038)<br>- FF CoQ10 levels were higher in pregnant women (0.603 vs 0.379 μg/ml, p = 0.044)<br>- Threshold for pregnancy > 0.255 μg/ml.
Lana Nazar Abdul-Razzaq et al., 2020, Case control [19]	117 women, 21 had a successful implantation, 96 had an implantation failure	31.1 ± 5.7	Total anti-oxidant capacity (TAC)	Implantation rate	**-** Higher TAC levels are correlated with implantation success (1.08 vs 0.55 mmol/L, p = 0.002)<br>**-** 50% of women with implantation success vs 6.1 % of women with implantation failure had sufficient TAC levels (> 1.3 mmol/L)<br>**-** 84.4% of women with implantation failure vs 40% of women with implantation success had low TAC levels (< 1 mmol/L)<br>**- T**he rest had borderline TAC levels between 1 and 1.3 mmol/L (9.1% vs 10%)
Zhengao Sun et al., 2017, Cohort [15]	48 women, 24 aged 23 - 28 years and 28 aged 35 - 46	- Young age group: 23 - 28<br>- Older age group: 35 - 46	Nicotine glucuronide, 4,5-dihydroorotic acid (4,5-DHOA), 5,6-dihydrouridine (5,6-DHU)	Oocyte quality	Nicotine glucuronide 4,5-DHOA and 5,6-DHU were negatively correlated with oocyte quality (p = 0.001, 0.007, 0.005)

Pelin Ocal et al., 2012, Case control [20]	50 women, 6 had PCOS, 28 had a partner with male factor infertility. 17 achieved clinical pregnancy and 33 didn't	- Pregnant: 29.7 ± 4.07<br>- Non-pregnant: 32.3 ± 4.7<br>- Not specified for male factor infertility subgroup	Homocysteine (Hcy)	Clinical pregnancy rate	- Higher FF homocysteine levels in women who couldn't achieve pregnancy in the male factor infertility subgroup (14.1 vs 9.9 μmol/L, p = 0.002)<br>- Threshold for pregnancy failure > 11.9 μmol/L

Supplementary Table S6. Studies on the effects of amino acids on IVF/ICSI outcomes in normo-ovulatory women.

Authors, year, study design	Participants	Age (years)	FF biomarker(s)	IVF/ICSI outcome(s)	Results
Jingyan Song et al., 2019, Cohort [14]	44 women divided into pregnancy loss group (n = 22) and control group (n = 22)	- pregnancy loss group: 29.7 ± 3.8,<br>- Control group: 30.3 ± 3.5	Phenylalanine, Leucine, Tryptophan	Recurrent pregnancy loss rate	FF levels of phenylalanine, leucine and tryptophan were downregulated in the pregnancy loss group (p = 0.0009, 0.0022, 0.0003)
Zhengao Sun et al., 2017, Cohort [15]	48 women, 24 aged 23 - 28 years and 28 aged 35 - 46	- Young age group: 23 - 28<br>- Older age group: 35 - 46	Maleylacetoacetic acid (MAAA), Rhazidigenine Nb-oxide	Oocyte quality	FF levels of MAAA and rhazidigenine Nb-oxide were negatively correlated with oocyte quality (p = 0.008, 0.04)

Supplementary Table S7. Studies on the effects of vitamins on IVF/ICSI outcomes in normo-ovula-tory women.

Authors, year, study design	Participants	Age (years)	FF biomarker(s)	IVF/ICSI outcome(s)	Results
Christofani Ekapatria et al., 2022, Cohort [21]	77 women, 39 with FF vitamin D levels over 13.7 ng/mL and 38 with levels under 13.7 ng/mL	20 - 35	Vitamin D	Fertilization rate, oocyte quality.	- Higher FF vitamin D levels in higher-quality oocytes (p = 0.01)<br>- No significant difference in fertilization rates between the two groups (71.8% vs 55.26%, p = 0.13)
Jingyan Song et al., 2019, Cohort [14]	44 women, divided into pregnancy loss group (n = 22) and control group (n = 22)	- pregnancy loss group: 29.7 ± 3.8<br>- Control group: 30.3 ± 3.5	Vitamin D, Lithocholic acid, 13-hydroxy-alpha-tocopherol (13'-HAT)	Recurrent pregnancy loss rate, oocyte quality	Lower FF vitamin D, Lithocholic acid and 13-hydroxy-alpha-tocopherol levels in pregnancy loss group compared to control group (p = 0.0017, 0.0008, 0.0019)

Funda Gode et al., 2019, Case control [22]	58 women split into two groups according to embryo quality:<br>- Group 1: high-quality<br>- Group 2: low-quality	- Group 1: 31.15 ± 4.56<br>- Group 2: 33.75 ± 4.46	Vitamins A, B6, D and E	Embryo quality, clinical pregnancy rates	- Higher vitamin A and B6 levels correlated with higher embryo quality (p = 0.017, 0.049)<br>- Vitamin D and E levels weren't associated with embryo quality (p = 0.999, 0.505)<br>- FF levels of all vitamins weren't significantly correlated with pregnancy rates (p = 0.830, 0.685, 0.971, 0.673)
Zhengao Sun et al., 2017, Cohort [15]	48 women, 24 aged 23 - 28 years and 28 aged 35 - 46	- Young age group: 23 – 28<br>- Older age group: 35 - 46	4-oxo-Retinoic acid	Oocyte quality	FF levels of 4-oxo-Retinoic acid were negatively correlated with oocyte quality (p = 0.03)

Supplementary Table S8. Studies on the effects of lipids on IVF/ICSI outcomes in normo-ovulatory women.

Authors, year, study design	Participants	Age (years)	FF biomarker(s)	IVF/ICSI outcome(s)	Results
Zhengao Sun et al., 2017, Cohort [15]	48 women, 24 aged 23 - 28 years and 28 aged 35 - 46	- Young age group: 23 - 28<br>- Older age group: 35 - 46	3 energy metabolism lipids:<br>- Triglyceride(18:1/24:0/20:5)<br>- Diacylglycerol (14:1/22:2)<br>- 3-hydroxynonanoyl-L-carnitine (3-HNC)<br>5 membrane phosopholipids:<br>- LysoPC(14:0)<br>- LysoPC(16:0)<br>- LysoPC(18:0)<br>- Phytosphingosine<br>- Phosphatidylcholine (PC)	Oocyte quality	- TG(18:1/24:0/20:5), DG(14:1/22:2), LysoPC(14:0), LysoPC(16:0), LysoPC(18:0) and phytosphingosine positively correlated with oocyte quality (p = 0.03, 0.04, 0.02, 0.03, 0.04, 0.02)<br>- 3-HNC and PC negatively correlated with oocyte quality (p = 0.04, 0.001)

Jingyan Song et al., 2019, Cohort [14]	44 women divided into pregnancy loss group (n = 22) and control group (n = 22)	- pregnancy loss group: 29.7 ± 3.8<br>- Control group: 30.3 ± 3.5	7 membrane phosopholipids:<br>- LysoPC(16:0)<br>- LysoPC(18:0)<br>- LysoPC(18:1)<br>- LysoPC(18:2)<br>- LysoPC(20:3)<br>- LysoPC(20:4)<br>- LysoPC(20:5)<br>3 fatty acids:<br>- Docosahexaen-oic acid (DHA)<br>- Linoleate<br>- Oleic acid	Recurrent pregnancy loss rate	- LysoPC(16:0), LysoPC(18:0), LysoPC(18:1), LysoPC(18:2), LysoPC(20:3), LysoPC(20:4) and LysoPC(20:5) were upregulated in the pregnancy loss group (p = 0.0073, 0.0007, 0.0068, 0.0091, 0.0012, 0.0009, 0.0023)<br>- DHA, linoleate and oleic acid were downregulated in the pregnancy loss group (p = 0.0008, 0.0028, 0.0052)
Bahareh Habibi et al., 2022, Cohort [12]	70 women, 51 with history of RIF and 19 con-trols. 19 of RIF group achieved pregnancy, 32 didn't	< 38	Prostaglandin E2 (PGE2)	Clinical pregnancy rate	FF PGE2 levels were higher in women who achieved pregnancy compared to women who didn't (87.31 vs 73.65 pg/ml, p < 0.0001), compared to a control group with-out a history of RIF (78.73 pg/ml)

Supplementary Table S9. Studies on the effects of miRNAs on IVF/ICSI outcomes in normo-ovula-tory women.

**Authors, year, study design**	**Participants**	**Age (years)**	**FF biomarker(s)**	**IVF/ICSI outcome(s)**	**Results**
Bahareh Habibi et al., 2022, Cohort [12]	70 women, 51 with history of re-current implanta-tion failure (RIF) and 19 controls. 19 of RIF group achieved preg-nancy and 32 didn't	< 38	miR-26b-5p<br>miR-34a-5p<br>miR-145-5p<br>miR-204-5p	Embryo quality, clini-cal pregnancy rate	- Upregulation of miR-26b-5p and downregulation of miR-34a-5p was significant in higher-quality embryos (p = 0.004, 0.02)<br>- Expression levels of miR-145-5p and miR-204-5p weren't signifi-cant for embryo quality (p = 0.8, 0.2)<br>- Upregulation of miR-26b-5p and downregulation of miR-34a-5p, miR-145-5p and miR-204-5p was significant in RIF women who achieved pregnancy (p = 0.031, 0.048, 0.037, 0.046)

References

1. Sun, X.; Jin, J.; Zhang, Y.L.; Ma, Y.; Zhang, S.; Tong, X. Decreased Histidine-Rich Glycoprotein and Increased Complement C4-B Protein Levels in Follicular Fluid Predict the IVF Outcomes of Recurrent Spontaneous Abortion. *Clin Proteomics* **2022**, *19*, doi:10.1186/S12014-022-09383-9.
2. Bastu, E.; Gokulu, S.G.; Dural, O.; Yasa, C.; Bulgurcuoglu, S.; Karamustafaoglu Balci, B.; Celik, C.; Buyru, F. The Association between Follicular Fluid Levels of Cathepsin B, Relaxin or AMH with Clinical Pregnancy Rates in Infertile Patients. *European Journal of Obstetrics and Gynecology and Reproductive Biology* **2015**, *187*, 30–34, doi:10.1016/J.EJOGRB.2015.02.009.
3. Atabakhsh, M.; Khodadadi, I.; … I.A.-… of reproduction & Activity of Matrix Metalloproteinase 2 and 9 in Follicular Fluid and Seminal Plasma and Its Relation to Embryo Quality and Fertilization Rate. *ncbi.nlm.nih.gov* **2018**.
4. Bilen, E.; Tola, E.N.; Oral, B.; Doguç, D.K.; Günyeli, İ.; Köse, S.A.; İlhan, I. Do Follicular Fluid Gelatinase Levels Affect Fertilization Rates and Oocyte Quality? *Arch Gynecol Obstet* **2014**, *290*, 1265–1271, doi:10.1007/S00404-014-3370-X.
5. Mostafa, M.; Faisal, M.; … N.M.-O.J. of O. Effect of Follicular Fluid Lactoferrin Level on Oocytes Quality and Pregnancy Rate in Intracytoplasmic Sperm Injection Cycles. *scirp.org* **2019**.
6. Jasimkadim, E.; Wasiti, E. Al; Qader, H. Concentration of R-Spondin 2 in the Follicular Fluid Is Correlated with Implantation Rate, Estrogen and Amphiregulin, in Iraqi Women Undergo ICSI. *academia.edu* **2020**.
7. Wu, Y.T.; Wang, T.T.; Chen, X.J.; Zhu, X.M.; Dong, M.Y.; Sheng, J.Z.; Xu, C.M.; Huang, H.F. Bone Morphogenetic Protein-15 in Follicle Fluid Combined with Age May Differentiate between Successful and Unsuccessful Poor Ovarian Responders. *Reproductive Biology and Endocrinology* **2012**, *10*, doi:10.1186/1477-7827-10-116.
8. Mehta, B.N.; Chimote, N.M.; Chimote, M.N.; Chimote, N.N.; Nath, N.M. Follicular Fluid Insulin like Growth Factor-1 (FF IGF-1) Is a Biochemical Marker of Embryo Quality and Implantation Rates in in Vitro Fertilization Cycles. *J Hum Reprod Sci* **2013**, *6*, 140–146, doi:10.4103/0974-1208.117171.
9. Celik, O.; Celik, E.; Yilmaz, E.; Celik, N.; Turkcuoglu, I.; Ulas, M.; Kumbak, B.; Aktan, E.; Ozerol, I. Effect of Ovarian Stimulation with Recombinant Follicle-Stimulating Hormone, Gonadotropin-Releasing Hormone Agonist and Antagonists, on Follicular Fluid Stem Cell Factor and Serum Urocortin 1 Levels on the Day of Oocyte Retrieval. *Arch Gynecol Obstet* **2013**, *288*, 1417–1422, doi:10.1007/s00404-013-2804-1.
10. Carpintero, N.L.; Suárez, O.A.; Mangas, C.C.; Varea, C.G.; Rioja, R.G. Follicular Steroid Hormones as Markers of Oocyte Quality and Oocyte Development Potential. *J Hum Reprod Sci* **2014**, *7*, 187–193, doi:10.4103/0974-1208.142479.
11. Yang, J.; Yang, X.; Yang, H.; Bai, Y.; Zha, H.; Jiang, F.; Meng, Y. Interleukin 6 in Follicular Fluid Reduces Embryo Fragmentation and Improves the Clinical Pregnancy Rate. *J Assist Reprod Genet* **2020**, *37*, 1171–1176, doi:10.1007/s10815-020-01737-2.
12. Habibi, B.; Novin, M.; Salehpour, S.; Novin, M.; Mohammadi Yeganeh, S.; Nazarian, H. Expression Analysis of Genes and MicroRNAs Involved in Recurrent Implantation Failure: New Noninvasive Biomarkers of Implantation. *Biomedical and Biotechnology Research Journal* **2022**, *Biomedical*, Article Journal, doi:10.4103/bbrj.bbrj_246_21.
13. Alzubaidy, H.; Alizzi, F.; Mossa, H. The Correlation between Follicular Fluid Levels of Progesterone and Anti-Müllerian Hormone and Pregnancy Rate in ICSI-Cycle. *researchgate.net* **2023**, doi:10.31533/pubvet.v14n6a592.1-6.
14. Song, J.; Wang, X.; Guo, Y.; Yang, Y.; Xu, K.; Wang, T.; Sa, Y.; Yuan, L.; Jiang, H.; Guo, J.; et al. Novel High-Coverage Targeted Metabolomics Method (SWATHtoMRM) for Exploring Follicular Fluid Metabolome

Alterations in Women with Recurrent Spontaneous Abortion Undergoing in Vitro Fertilization. *Sci Rep* **2019**, *9*, doi:10.1038/S41598-019-47370-7.

15. Sun, Z.; Wu, H.; Lian, F.; Zhang, X.; Pang, C.; Guo, Y.; Song, J.; Wang, A.; Shi, L.; Han, L. Human Follicular Fluid Metabolomics Study of Follicular Development and Oocyte Quality. *Chromatographia* **2017**, *80*, 901–909, doi:10.1007/s10337-017-3290-6.
16. Mutaz, S.; Ahmeid, M.B.; Ch, B. Correlation between Follicular Fluid Leptin and the Pregnancy Rate in Women Who Underwent ICSI. *iasj.net* **2017**, *22*, 248–253.
17. Buyuk, E.; Asemota, O.A.; Merhi, Z.; Charron, M.J.; Berger, D.S.; Zapantis, A.; Jindal, S.K. Serum and Follicular Fluid Monocyte Chemotactic Protein-1 Levels Are Elevated in Obese Women and Are Associated with Poorer Clinical Pregnancy Rate after in Vitro Fertilization: A Pilot Study. *Fertil Steril* **2017**, *107*, 632-640.e3, doi:10.1016/J.FERTNSTERT.2016.12.023.
18. Akarsu, S.; Gode, F.; Isik, A.Z.; Dikmen, Z.G.; Tekindal, M.A. The Association between Coenzyme Q10 Concentrations in Follicular Fluid with Embryo Morphokinetics and Pregnancy Rate in Assisted Reproductive Techniques. *J Assist Reprod Genet* **2017**, *34*, 599–605, doi:10.1007/S10815-017-0882-X.
19. Abdul-Razzaq, L.; Salih, K.; Update, B.A.-M.-M. Evaluation of Total Antioxidant Capacity in Serum and Follicular Fluid of Women Undergoing ICSI and Its Association with Implantation Failure. *academia.edu* **2020**.
20. Ocal, P.; Ersoylu, B.; Cepni, I.; Guralp, O.; … N.A.-J. of assisted; 2012, undefined The Association between Homocysteine in the Follicular Fluid with Embryo Quality and Pregnancy Rate in Assisted Reproductive Techniques. *Springer* **2012**.
21. Ekapatria, C.; Hartanto, B.; Wiryawan, P.; Tono, D.; Maringan Diapari Lumban, T.; Meita, D.; Arief, B.; Johanes Cornelius, M. The Effects of Follicular Fluid 25(OH)D Concentration on Intrafollicular Estradiol Level, Oocyte Quality, and Fertilization Rate in Women Who Underwent IVF Program. *Journal of Obstetrics and Gynecology of India* **2022**, *72*, 313–318, doi:10.1007/S13224-021-01615-6.
22. Gode, F.; Akarsu, S.; Dikmen, Z.; … B.T.-G.O.& The Effect Follicular Fluid Vitamin A, E, D and B6 on Embryo Morphokinetics and Pregnancy Rates in Patients Receiving Assisted Reproduction. *gorm.com.tr* **2019**, *25*, 89–95, doi:10.21613/GORM.2018.860.
