# Supplementary material for: Associations Between Follicular Fluid Biomarkers and IVF/ICSI Outcomes in Normo-Ovulatory Women—A Systematic Review
Source: Biomolecules. 2025 Mar 20;15(3):443. doi: 10.3390/biom15030443 (PMC11940193; doi:10.3390/biom15030443)
Supplement: Supplementary file 1 [file biomolecules-15-00443-s001.zip › S7. Abbreviations.pdf]

Abbreviations

The following abbreviations are used in this manuscript:

FF	follicular fluid
IVF	*in vitro* fertilization
ICSI	intracytoplasmic sperm injection
ARTs	Assisted reproductive techniques
miRNAs	microRNAs
PCOS	polycystic ovary syndrome
PRISMA	Preferred Reporting Items for Systematic reviews and Meta-Analyses
PROSPERO	The International Prospective Register of Systematic Reviews
NOS	Newcastle-Ottawa Scale
TCs	theca cells
GCs	granulosa cells
COC	cumulus oocyte complex
HRG	Histidine-rich glycoprotein
MII	metaphase II, mature oocytes
MMP-2	Matrix metalloproteinase-2
MMP-9	Matrix metalloproteinase-9
AREG	Amphiregulin
BMP-2	Bone morphogenic protein-2
BMP-4	Bone morphogenic protein-4
BMP-10	Bone morphogenic protein-10
BMP-15	Bone morphogenic protein-15
IGF-1	Insulin-like growth factor-1
IGF-1R	Insulin-like growth factor-1 receptor
SCF	Stem cell factor
E2	Estradiol
DHEAS	Dehydroepiandrosterone sulfate
25-HC	25-hydroxycholesterol
DOC	Deoxycorticosterone
AMH	Anti-mullerian hormone
IL-6	Inetrleukin-6
IL-6R	Interleukin-6 receptor
IL-11	Inetrleukin-11
IL-1β	Inetrleukin-1β
TNF-α	tumor necrosis factor alpha
MCP-1	Monocyte chemotactic protein-1
CoQ10	Coenzyme Q10
TAC	Total antioxidant capacity
4,5-DHOA	4,5-dihydroorotic acid
5,6-DHU	5,6-dihydrouridine
Hcy	Homocysteine
MAAA	Maleylacetoacetic acid
4-oxo-RA	4-oxo-Retinoic acid
13′-HAT	13′-hydroxy-alpha-tocopherol
EGF	Epidermal growth factor
TGF-β	Transforming growth factor-β
O.C	oocyte count
O.Q	oocyte quality
F.R	fertilization rate

E.Q	embryo quality
I.R	implantation rate
P.R	pregnancy rate
M.R	miscarriage or pregnancy loss rate
L.B.R	live birth rate
RPL	recurrent pregnancy loss
BMI	Body mass index
ETC	electron transport chain
RNA	Ribonucleic acid
tRNA	transfer RNA
mRNA	messenger RNA
DNA	Deoxyribonucleic acid
rDNA	recombinant DNA
3-HNC	3-hydroxynonanoyl-L-carnitine
LysoPCs	Lysophosphatidylcholines
PC	phosphatidylcholine
LPA	lysophosphatidic acid
DHA	Docosahexaenoic acid
AA	arachidonic acid
PGE2	Prostaglandin E2
PGF2	Prostaglandin F2
RIF	Repeated implantation failure
BFB	blood-follicle barrier
ECM	extracellular matrix
MAC	membrane attack complex
ELISA	enzyme-linked immunosorbent assay
CLIA	chemiluminescent immunoassay
CL-MIA	CL-based microarrays
PCR	polymerase chain reaction
RT-qPCR	quantitative real-time PCR
WB	western blot
LC-MS	liquid chromatography with mass spectrometry
SWATHtoMRM	Sequential Window Acquisition of all Theoretical Mass Spectra to Multiple-Reac-tion Monitoring
HBEGF	heparin-binding EGF-like growth factor
EGFR	epidermal growth factor receptor
VEGF	vascular endothelial growth factor
VE-cadherin	vascular endothelial cadherin
GDF9	growth differentiation factor 9
GH	growth hormone
GHR	growth hormone receptor
COX-1	cyclooxygenase 1
COX-2	cyclooxygenase 2
LDLR	low-density lipoprotein receptor
PR	Progesterone receptor
ER	estrogen receptor
FSHR	Follicle-stimulating hormone receptor
ROS	Reactive Oxygen Species
DAMPs	damage-associated molecular patterns
DHODH	dihydroorotate dehydrogenase
CβS	cystathionine beta-synthase
VDR	Vitamin D receptor
SOD	superoxide dismutase
CAT	catalase
GR	glutathione reductase

GPx	glutathione peroxidase
RA	retinoic acid
4-OH-RA	4-hydroxy-retinoic acid
NTDs	neural tube defects
TGs	Triglycerides
DGs	Diglycerides
3-HNC	3-hydroxynonanoyl-L-carnitine
MGs	Monoglycerides
PKA	protein kinase A
PKC	protein kinase C
PPOS	progestin-primed ovarian stimulation
MPA	medroxyprogesterone 17-acetate
PLA2	phospholipase A2
ATX	autotaxin
FST	follistatin
S1P	Sphingosine-1 Phosphates
P1P	Phytosphingosine-1-phosphate
EPA	Eicosapentaenoic acid
DHA	docosahexaenoic acid
3′-UTR	3′ untranslated region
5′-UTR	5′ untranslated region
PmmR	putative microRNA-microRNA regulations
DIA	data-independent acquisition
IVS	*in vitro* supplementation
IFN-γ	interferon gamma
CAMK1D	calcium/calmodulin dependent protein kinase ID
FSH	follicle-stimulating hormone
LAT1	L-type amino acid transporter 1
15-PGDH	15-hydroxyprostaglandin dehydrogenase
Th1	T helper 1 cell
Th2	T helper 2 cell
Tregs	regulatory T cells
StAR	steroidogenic acute regulatory protein
