# Supplementary material for: Associations Between Follicular Fluid Biomarkers and IVF/ICSI Outcomes in Normo-Ovulatory Women—A Systematic Review
Source: Biomolecules. 2025 Mar 20;15(3):443. doi: 10.3390/biom15030443 (PMC11940193; doi:10.3390/biom15030443)
Supplement: Supplementary file 1 [file biomolecules-15-00443-s001.zip › S3. Search phrases.pdf]

The three following search phrases were used to parse the PubMed and Scopus databases, and the Google Scholar search engine, and resulted in 122, 70 and 235 articles, respectively as of October 1st 2024. The “include citations” option was turned off in Google Scholar results, and although publish dates were specified to be after 2009, i.e. starting January 1st 2010, the Google Scholar search still resulted in 30 articles that were published before 2010. Those articles were filtered out.

PubMed

((Hormone*[ti] OR Androgen*[ti] OR Progesterone*[ti] OR Estradiol*[ti] OR Estrone*[ti] OR DHEA*[ti] OR Testosterone*[ti] OR Androstenedione*[ti] OR Androsterone*[ti] OR “anti-mullerian hormone”[ti] OR AMH[ti]) OR ("Vitamin D"[ti] OR "25(OH)D"[ti] OR “Hydroxyvitamin D”[ti] OR Cholecalciferol[ti] OR Hydroxycholecalciferol*[ti] OR Dihydrotachysterol*[ti] OR "Vitamin D"[MeSH] OR Cholecalciferol[MeSH] OR Hydroxycholecalciferols[MeSH] OR Ergocalciferols[MeSH] OR Dihydrotachysterol[MeSH]) OR (Cytokine*[ti] OR interleukin*[ti] OR "tumor necrosis factor*"[ti] OR TNF[ti] OR Chemokine*[ti] OR Lymphokine*[ti]) OR (MicroRNA*[MeSH] OR MicroRNA*[ti] OR “Micro RNA*”[ti] OR miRNA*[ti] OR “RNA, Micro”[ti] OR pri-miRNA*[ti] OR “pri miRNA*”[ti] OR stRNA*[ti] OR “Small Temporal RNA”[ti] OR pre-miRNA*[ti] OR “pre miRNA*”[ti] OR siRNA*[ti] OR "small interfering RNA*"[ti] OR RNA[ti]) OR (Protein*[ti] OR proteomic*[ti] OR polypeptide*[ti] OR glycoprotein*[ti] OR albumin*[ti] OR Fibronectin*[ti] OR Transferrin*[ti] OR globulin*[ti] OR macroglobulin*[ti] OR antitrypsin*[ti] OR IGF[ti] OR AMH[ti] OR antimullerian*[ti] OR "Bone morphogenetic protein*"[ti] OR "Kit ligand*"[ti] OR "stem cell factor*"[ti] OR “antioxidant enzyme*”[ti]) OR (Metaboli*[ti] OR Metabolomic*[ti] OR DHEA*[ti] OR Dehydroepiandrosterone*[ti] OR Glutathione*[ti]) OR (Gluco*[ti] OR Dextro*[ti] OR sugar*[ti] OR carbohydrate*[ti] OR glyco*[ti] OR glycemi*[ti]) OR ((Growth*[ti] AND factor*[ti]) OR "Growth factor*"[ti] OR (Growth*[ti] AND Hormone*[ti]) OR “Growth hormone*”[ti] OR EGF[ti] OR TGF[ti] OR IGF[ti] OR FGF[ti] OR VEGF[ti] OR PDGF[ti]) OR (“Reactive oxygen species”[ti] OR ROS[ti] OR “Oxygen Radical*”[ti] OR “Radical, Oxygen”[ti] OR “Active Oxygen Species”[ti] OR “Reactive Oxygen Intermediates”[ti] OR “Active Oxygen”[ti] OR “Oxygen, Active”[ti] OR “Oxygen Species, Reactive”[ti] OR “Oxygen Radicals”[ti] OR “Pro-Oxidant*”[ti] OR “Pro Oxidant*”[ti])) AND ("pregnancy rate*"[ti] OR "implantation rate*"[ti] OR "implantation failure"[ti] OR "fertilization rate*"[ti] OR “fertilization failure”[ti] OR "oocyte quality"[ti] OR abortion*[ti] OR “live birth rate”) AND (follicular*[tiab] OR “follicular fluid”[MeSH]) AND 2010:2024[pdat] NOT (review[ti] OR "polycystic ovarian syndrome"[ti] OR "polycystic ovary syndrome"[ti] OR PCOS[ti] OR endometriosis[ti])

Scopus

((TITLE(Hormone* OR Androgen* OR Progesterone* OR Estradiol* OR Estrone* OR DHEA* OR Testosterone* OR Androstenedione* OR Androsterone* OR “anti-mullerian hormone” OR AMH)) OR (TITLE("Vitamin D" OR "25?OH?D" OR "Hydroxyvitamin D" OR Cholecalciferol OR Hydroxycholecalciferol* OR Dihydrotachysterol*) OR KEY("Vitamin D" OR Cholecalciferol OR Hydroxycholecalciferols OR Ergocalciferols OR Dihydrotachysterol)) OR (TITLE(Cytokine* OR interleukin* OR {tumor necrosis factor} OR TNF OR Chemokine* OR Lymphokine*)) OR (KEY(MicroRNA) OR TITLE(MicroRNA* OR "Micro RNA" OR miRNA OR "RNA, Micro" OR pri-miRNA* OR "pri miRNA" OR stRNA* OR "Small Temporal RNA" OR pre-miRNA* OR "pre miRNA" OR siRNA* OR "small interfering RNA" OR RNA)) OR (TITLE(Protein* OR proteomic* OR polypeptide* OR glycoprotein* OR albumin* OR Fibronectin* OR Transferrin* OR globulin* OR macroglobulin* OR antitrypsin* OR IGF OR AMH OR antimullerian* OR "Bone morphogenetic protein" OR "Kit ligand" OR "stem cell factor" OR "antioxidant enzyme")) OR (TITLE(Metaboli* OR Metabolomic* OR DHEA* OR Dehydroepiandrosterone* OR Glutathione*)) OR (TITLE(Gluco* OR Dextro* OR sugar* OR carbohydrate* OR glyco* OR glycemi*)) OR (TITLE((Growth* AND factor*) OR "Growth factor" OR "Growth factors" OR (Growth* AND Hormone*) OR "Growth hormone" OR "Growth hormones" OR EGF OR TGF OR IGF OR FGF OR VEGF OR PDGF)) OR (TITLE("Reactive oxygen species" OR ROS OR "Oxygen Radical" OR "Oxygen Radicals" OR "Radical, Oxygen" OR "Active Oxygen Species" OR "Reactive Oxygen Intermediates" OR "Active Oxygen" OR "Oxygen, Active" OR "Oxygen Species, Reactive" OR "Pro-Oxidant" OR "Pro-Oxidants" OR "Pro Oxidant" OR "Pro Oxidants"))) AND TITLE("oocyte quality" OR abortion OR ((pregnancy OR implantation OR fertilization OR "live birth") AND (rate* OR failure*))) AND (TITLE(follicular*)OR ABS(follicular*) OR KEY {follicular fluid}) AND PUBYEAR > 2009 AND NOT(TITLE(review OR endometriosis OR PCOS OR {polycystic ovarian syndrome} OR {polycystic ovary syndrome}))

Google Scholar

allintitle:((“oocyte quality” | abortion | ((pregnancy | implantation | fertilization | “live birth”) (rate | rates | failure | failures))) (follicular)) after:2009 -intitle:review -intitle:endometriosis -intitle:PCOS -intitle:“polycystic ovarian syndrome” -intitle:“polycystic ovary syndrome”
